# Uni- and crossmodal refractory period effects of event-related potentials provide insights into the development of multisensory processing

**DOI:** 10.3389/fnhum.2014.00552

**Published:** 2014-07-28

**Authors:** Jessika Johannsen, Brigitte Röder

**Affiliations:** ^1^Neuropediatrics, University Medical Center Hamburg-EppendorfHamburg, Germany; ^2^Biological Psychology and Neuropsychology, University of HamburgHamburg, Germany

**Keywords:** auditory event-related potentials, visual event-related potentials, refractory periods, crossmodal, multisensory processing, development, unimodal

## Abstract

To assess uni- and multisensory development in humans, uni- and crossmodal event-related potential (ERP) refractory period effects were investigated. Forty-one children from 4 to 12 years of age and 15 young adults performed a bimodal oddball task with frequent and rare visual and auditory stimuli presented with two different interstimulus intervals (ISIs). Amplitudes of the visual and auditory ERPs were modulated as a function of the age of the participants, the modality of the preceding stimulus (same vs. different) and the preceding ISI (1000 or 2000 ms). While unimodal refractory period effects were observed in all age groups, crossmodal refractory period effects differed among age groups. Early crossmodal interactions (<150 ms) existing in the youngest age group (4–6 years) disappeared, while later crossmodal interactions (>150 ms) emerged with a parietal topography in older children and adults. Our results are compatible with the intersensory differentiation and the multisensory perceptual narrowing approach of multisensory development. Moreover, our data suggest that uni- and multisensory development run in parallel with unimodal development leading.

## Introduction

Event-related potentials (ERPs) have been used to assess the neuronal mechanisms of multisensory interactions (Stein and Meredith, 1993; Driver and Noesselt, 2008; Senkowski et al., 2008) and to investigate neurocognitive development (e.g., Courchesne, 1978; De Haan and Nelson, 1997; Molfese and Molfese, 2000; Nelson and Monk, 2001). In the past, multisensory development has mostly been evaluated with behavioral techniques (for a recent review see Bremner et al., 2012). Different views on how multisensory functions emerge have been proposed: While the hierarchical development view (=intersensory integration view, sometimes linked to the constructivism view of development) assumes that the modality systems initially develop in isolation and are linked only later in life (Piaget, 1952), the intersensory differentiation view proposes that multisensory interactions exist already at birth, and are then shaped by experience in the first months of life (Gibson, 1969, 1984; Maurer, 1993). Moreover, Lewkowicz and Ghazanfar (2009) postulated that the development of multisensory processing emerges through regressive events, leading to a loss of multisensory abilities as a function of environmental experiences (multisensory perceptual narrowing view).

One approach to study the neural correlates of multisensory development is to record ERPs. Brett-Green et al. (2008) compared ERPs to bimodal stimuli and to the sum of ERPs elicited by the comprising unimodal events. They detected multisensory interactions between the auditory and the somatosensory system in children aged 6 to 13 years. A similar protracted developmental time course of multisensory development was suggested by the results of Brandwein et al. (2010), who reported a mature pattern of multisensory ERP effects not before the age of 14 years of age. Since the ERP approach chosen by these authors has been criticized (Gondan et al., 2005), we decided to adapt a paradigm that has fruitfully been used to study unisensory development (Cheour and Näätänen, 1998; Coch et al., 2005): When two stimuli are presented in succession, the amplitudes of ERPs elicited by the second stimulus are decreased as a function of the interstimulus interval (ISI). The time it takes the ERP amplitudes to fully recover is called the refractory period (Neville et al., 1983; Ritter et al., 1992). ERP refractory periods within a sensory system have been interpreted as an index of the excitability of the neural networks contributing to a particular ERP (Neville et al., 1983; Ritter et al., 1992). Unisensory ERP refractory periods have been used in humans to study the development of sensory systems (Cheour and Näätänen, 1998; Coch et al., 2005) and to understand the functional changes of sensory cortices following sensory loss (Neville et al., 1983). Coch et al. (2005) used auditory and visual refractory periods (with ISIs between 360 and 2000 ms) to investigate the developmental trajectory of the auditory and visual system between the age of 6 and 8 years. They reported adult-like patterns of unimodal refractory period effects in school-age children.

To date, crossmodal ERP refractory periods have not been systematically investigated and ERP studies that directly compare uni- and crossmodal refractory period effects in the same individuals and in populations aged from early childhood through adulthood are still missing. In adults, Fruhstorfer (1971) found an amplitude recovery of the vertex ERP when in a regular train of stimuli of the same modality (auditory or tactile) a stimulus of a different modality (tactile and auditory, respectively) was interposed. Furthermore, Davis et al. (1972) measured vertex ERP amplitudes to auditory, visual and tactile stimuli which were preceded by either a stimulus of the same or of a different modality with an ISI of 500 ms. They reported crossmodal refractory period effects for all modality pairings. The development of uni- vs. crossmodal refractory effects has, to our knowledge, not been investigated yet. Such studies would allow comparing the time course of both uni- and crossmodal development within the same paradigm. Moreover, due to the low demands required to perform the task, the very same paradigm is suitable for the use across a large age range. In our paradigm, healthy children (4–12 years of age) and adults were engaged in a simple bimodal oddball task with frequent and rare auditory and visual stimuli. ISIs (1000 and 2000 ms) between two consecutive stimuli of the same or of different modalities were systematically manipulated and uni- and crossmodal refractory period effects for both auditory and visual ERPs were assessed. We expected ERP amplitudes to vary as a function of ISI both within and across modalities. It was assumed that in the course of development, the distribution of refractory period effects on the scalp becomes more focused to a reduced number of electrodes. A hierarchical developmental view would predict a sequential emergence of uni- and crossmodal refractory effects. By contrast, a differentiation developmental view would predict a parallel emergence of uni- and crossmodal refractory effects.

## Materials and methods

### Participants

Forty one children (age group 1: 4–6 years, mean age 5.9 years, *n* = 15, 9 females, 6 males; age group 2: 7–9 years, mean age 8.4 years, *n* = 13, 8 females, 5 males; age group 3: 10–12 years, mean age 11.2 years, *n* = 13, 8 females, 5 males) and 15 adults (19–39 years, mean age 26 years, 7 females, 8 males) participated. The adults were all students of the University of Hamburg. The participants had no history of neurological, language, speech, or articulatory disease. All participants or the parents of the participating children reported normal hearing and normal or corrected-to-normal vision. Prior to the start of the experiment the parents and adult participants gave informed consent and children were asked to agree to participate in the study. In addition, children were tested with (i) <12 years. Colored progressive matrices (CPM, by Raven), (ii) ≥12 years. Standard progressive matrices (SPM, by Raven) and the motor-free visual perception test (MVPT-3, by Colarusso and Hammill) to confirm normal intelligence and normal visual perception. These data are not reported here, but all children included in the final analyses fell within the normal range. Parents received a reimbursement for travel costs and other costs associated with participating. Adult participants received course credit or were compensated with 7 €/h. The experiment was conducted according to the guidelines laid down in the Declaration of Helsinki and the study was approved by the ethics committee, Medical Association Hamburg, Nr. 2653. Twelve additional children (7 in age group 1, 3 in age group 2, and 2 in age group 3) and 3 additional adults were tested, but later excluded because of excessive artifacts in their EEG recordings.

### Stimuli and procedure

Stimuli consisted of frequently presented standard tones [90% of the auditory stimuli; white noise bursts, duration 50 ms, 70 dB(A)] and frequently presented visual standard stimuli (90% of the visual stimuli; 10 × 10 checkerboard square, visual angle: 5°, duration: 50 ms). Additionally, rarely presented deviant sounds (10% of the auditory stimuli; animal sounds, duration: 400 ms, 70 dB) and rarely presented deviant pictures (10% of the visual stimuli; pictures of animal, 5° of visual angle for the longest (horizontal or vertical) size, duration: 50 ms) were used as target stimuli. These target stimuli were sounds or pictures of animals and were taken from a Multimodal Stimulus Set (Schneider et al., 2008). Schneider et al. had created the animal sounds by selecting characteristic sounds of natural objects out of 12 sound effect CDs (100 Spectacular Sound FX, Mediaphon, Leinfelden-Echterdingen, Germany) and the visual animal stimuli by selecting color photographs from a pool of pictures of a digital photo database (Hemera Photo Objects, Vol. 1, Hemera, Hull, Canada). Auditory and visual stimuli were presented in a randomized order. Six blocks in children and 12 blocks in adults, each lasting approximately 3 min were run. Each block consisted of 112 stimuli including at least one deviant stimulus. The first two stimuli of a block were randomly selected from one modality; they were not included in the analyses. A modality switch occurred in about half of the trials. Auditory and visual stimuli were presented with equal probability with an ISI of 1000 ms or 2000 ms. No more than four of the same ISI appeared in a sequence, all deviants were followed by an ISI of 2000 ms. The design of the study resulted in eight different stimulation conditions listed in Table 1. The task of the participants was to press a response button with either the right or the left hand as quickly as possible to each deviant stimulus irrespective of its modality. Prior to the start of the EEG recording the experiment was explained and one practice block consisting of a random sequence of auditory and visual stimuli (60 standard stimuli and 6 deviants) was run.

**Table 1:** **Characteristics of the eight different stimulation conditions**.

**Stimulus name**	**Preceding stimulus**	**Interstimulus interval**	**Stimulus eliciting the analyzed ERP**	**Condition**
asa	Auditory (a)	Short (s)	Auditory (a)	Auditory, unimodal, short ISI
ala	Auditory (a)	Long (l)	Auditory (a)	Auditory, unimodal, long ISI
vsa	Visual (v)	Short (s)	Auditory (a)	Auditory, crossmodal, short ISI
vla	Visual (v)	Long (l)	Auditory (a)	Auditory, crossmodal, long ISI
vsv	Visual (v)	Short (s)	Visual (v)	Visual, unimodal, short ISI
vlv	Visual (v)	Long (l)	Visual (v)	Visual, unimodal, long ISI
asv	Auditory (a)	Short (s)	Visual (v)	Visual, crossmodal, short ISI
alv	Auditory (a)	Long (l)	Visual (v)	Visual, crossmodal, long ISI

*Each condition is characterized by the preceding stimulus (first letter, auditory vs. visual), the interstimulus interval (second letter, short vs. long ISI) and the stimulus eliciting the analyzed ERP (third letter, auditory vs. visual) leading to stimulus names*.

The children were comfortably seated in a dimly lit, sound-attenuated, and electrically shielded chamber. They were instructed not to move their eyes from the fixation cross at the center of the monitor during a block. Parents attended the experiment room if the children asked for. Visual and auditory stimuli were presented on a computer screen and from a central loudspeaker, respectively, both located at a distance of 91.5 cm from the participants. Gaze direction was monitored by an experimenter via an infrarot camera.

### Data acquisition

**Figure 1:**
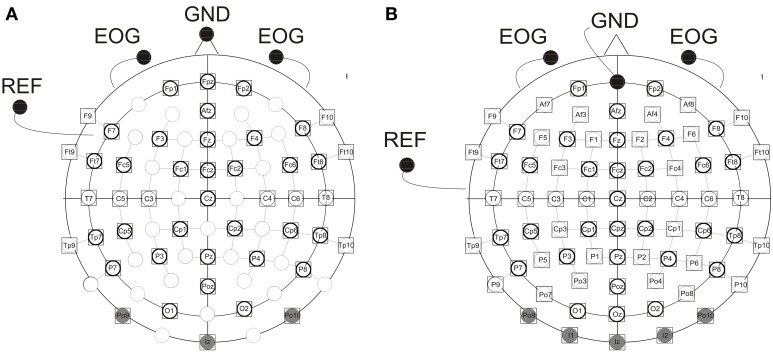
**Electrode montage with (A) 46 (children) and (B) 73 (adults) active electrodes, EOG, vertical electrooculogram recorded by two active elecrodes beneath the right and left eye; GND, ground electrode [nose tip (children), Fpz (adults)]; REF, reference electrode on the left ear lobe**. Electrodes shaded in gray were excluded from further analyses due to artifacts. Electrodes marked by a prominent circle were included in the statistical analyses.

EEG was recorded with 46 (children) and 73 (adults) active electrodes (ActiCap, Brain Products, Gilching, Germany) mounted in an elastic cap (Easycap, Falk Minow Services, Herrsching, Germany, see Figure 1). A lower number of electrodes was used in children because of their smaller heads. The EEG was recorded with a time constant of 10 s. Using BrainAmp amplifiers (Brain Products, Munich, Germany) the EEG signal was sampled at 5000 Hz, filtered online with a bandpass of 0.016 to 250 Hz and was then down sampled online to a sample rate of 500 Hz. These data were stored on a hard disk. Electrode positions were arranged according to the international 10–10 system. The left earlobe was used as reference electrode, offline a linked earlobe reference was calculated. The vertical EOG was recorded with two active electrodes beneath the right and the left eye against the common reference. Recordings from the electrodes F9 and F10 were used for the offline calculation of the horizontal EOG (HEOG). For each participant ERPs were averaged at each electrode site over an epoch of 700 ms (200 ms pre- to 500 ms post stimulus onset). ERPs were separately analyzed for each stimulus modality (auditory vs. visual) and were averaged as a function of the modality, of the preceding stimulus (same vs. different) and the preceding ISI (1000 vs. 2000 ms). Epochs following deviant stimuli were not included. Trials containing eye movements artifacts (HEOG and VEOG) exceeding ± 100 (children) or ± 80 (adults) μV relative to the absolute difference between any two sample points within the epoch (500 ms post stimulus) and other artifacts (a voltage exceeding ± 140 (children) or ± 120 (adults) μV at any electrode relative of any two sample points) were eliminated semiautomatically. Due to noisy recordings at the caudal electrodes (PO9, Iz, PO10) in a large number of the participants, these electrodes were excluded from further analyses. Only participants with a minimum of 25 (children) or 50 (adults) artifact free trials for each condition were included in the statistical analyses. Fourteen additional participants were excluded due to this criterion (see section Participants). The EEG data were acquired in the lab of the Biological Psychology and Neuropsychology, University of Hamburg, Germany.

### Data analyses

Based on earlier reports, and on visual inspection of the grand averages, mean amplitudes were calculated for the following time windows: Auditory ERPs: children: 90–150 ms, 150–250 ms, and 260–340 ms, adults: 70–120 ms, 140–250 ms, and 260–340 ms; visual ERPs: children: 100–150 ms, 150–220 ms, and 260–340 ms, adults: 90–140 ms, 140–190 ms, and 260–340 ms. Mean amplitudes of both visual and auditory ERPs were separately submitted to analyses of variance (ANOVA, using SPSS Software). The between-subject factor was Age group (age group 1, age group 2, age group 3, adults) and within-subject factors included Modality Transition (MT) (unimodal vs. crossmodal), ISI (short vs. long), Hemisphere (H) (right vs. left), Anterior Posterior (AP) (anterior, posterior), and Electrodes (E) (Fp1, F3, F7, FC1, FC5, FT7 vs. Fp2, F4, F8, FC2, FC6, FT8 vs. CP1, CP5, TP7, P3, P7, O1 vs. CP2, CP6, TP8, P4, P8, O2, see Figure 1). For midline electrodes an additional ANOVA was run, using three repeated measurement factors: Modality Transition (MT) (unimodal vs. crossmodal), ISI (short vs. long), and Electrodes (E) (Fpz (children), AFz, Fz, FCz, Cz, Pz, POz). In a second analysis, effects in the four different age groups were analyzed separately. Whenever, the interaction of factors MT and ISI and one of the topographic factors reached significance (*p* ≤ 0.05), the refractory period effect was tested at each single electrode separately for the uni- and crossmodal condition (paired *t*-tests, two-tailed). Topographic maps were created in Brain Vision Analyzer (Brain Products) using a linear interpolation algorithm. Finally, the ANOVA performed on the reaction times included the between-subject factor Age group (age group 1, age group 2, age group 3, adults) and the within-subject factors Modality of the current stimulus (visual vs. auditory), Modality Transition (MT) (unimodal vs. crossmodal) and ISI (short vs. long). Huynh/Feldt-corrected *p*-values, but uncorrected degrees of freedom, are reported when appropriate.

## Results

### Behavioral data

Five children in age group 1 were excluded from the behavioral data analyses, because they failed pressing the button all together. The remaining children detected 94% (*SE* = 1.4) and the adults detected 96% (*SE* = 3.8) of the deviants. Detailed results of the detection rates in each age group and for each modality are reported in Table 2. Because EEG data of these participants did not show any obvious difference to the other participants, EEG data of the five children without behavioral data were included in the statistical analyses.

**Table 2:** **Detection rates of deviant targets and standard error (SE) in each age group and each modality**.

	**Group 1 (4–6 years)**			**Group 2 (7–9 years)**			**Group 3 (10–12 years)**			**Adults**		
	**Total**	**Auditory**	**Visual**	**Total**	**Auditory**	**Visual**	**Total**	**Auditory**	**Visual**	**Total**	**Auditory**	**Visual**
In %	85.28	85.47	85.08	96.37	95.88	96.85	98.07	98.2	97.94	96	96.67	95.33
SE	6	6.3	6.1	1.4	2	1.3	1.6	0.9	1.6	3.8	1.9	7.5

The ANOVA performed on the reaction times revealed a main effect of Modality [*F*_(1, 47)_ = 99.6, *p* < 0.001] and significant interactions of ISI × MT × Age group [*F*_(3, 47)_ = 3.56, *p* < 0.05] and Modality × ISI × MT [*F*_(1, 47)_ = 5.91, *p* < 0.05]. These results were due to (i) faster RT for the visual than for the auditory targets across the groups and (ii) the influence of the preceding ISI, the preceding modality and the age of the participant on reaction times. RT to both visual and auditory stimuli decreased with age, with slowest RT in the youngest age group, and fastest RT in adults. Only for the visual stimuli, RT depended on the preceding ISI and the preceding modality, but these effects varied by the age group [ISI × MT × Age group, *F*_(3, 47)_ = 2.85, *p* < 0.05]. In the analyses conducted separately for each age group, no significant main effects and interactions were found in age group 1. Significant main effects for Modality [*F*_(1, 12)_ = 25.74, *p* < 0.001] and for MT [*F*_(1, 12)_ = 5.071, *p* < 0.05] in age group 2 and a significant main effect for Modality [*F*_(1, 12)_ = 52.17, *p* < 0.001]) in age group 3 were revealed. In adults, the ANOVA yielded a significant main effect for Modality [*F*_(1, 14)_ = 89.44, *p* < 0.001] and a marginally significant interaction of Modality, MT and ISI [*F*_(1, 12)_ = 3.9, *p* < 0.1]. For detailed results of average reaction times in each age group and each modality see Table 3.

**Table 3:** **Average reaction times (RT) and standard error (SE) for detecting deviant targets, separately for each age group, each modality and separately for all children and adults**.

	**AsAd**	**AlAd**	**VsAd**	**VlAd**	**VsVd**	**VlVd**	**AsVd**	**AlVd**
**AGE GROUP 1 (4–6 YEARS)**								
Average RT (ms)	883.97	875.29	912.15	898.86	778.06	826.01	870.91	766.25
SE	13.85	50.91	17.6	43.49	4.39	15.29	3.61	4.17
**AGE GROUP 2 (7–9 YEARS)**								
Average RT (ms)	796.86	798.61	747.69	800.66	681.02	675.58	655.72	649.33
SE	12.15	44.65	15.44	38.14	3.85	13.41	3.17	3.66
**AGE GROUP 3 (10–12 YEARS)**								
Average RT (ms)	695.11	691.57	712.88	703.71	588.46	601.71	566.76	584.18
SE	36.5	23.55	32.72	51.82	70.07	62.13	59.2	80.89
**AGE GROUP 1–3**								
Average RT (ms)	784.31	781.26	780.81	792.93	674.55	690.69	683.37	658.28
SE	54.57	53.27	61.44	56.33	54.73	65.99	90.28	53.26
**ADULTS**								
Average RT (ms)	692.98	667.49	669.26	669.28	543.83	563.31	544.96	535.29
SE	36.57	29.21	33.88	27.89	3.95	27.09	12.11	3.16
	**All auditory deviants**				**All visual deviants**			
**AGE GROUP 1–3**								
Average RT (ms)	784.83				676.72			
SE	5.62				13.95			
**ADULTS**								
Average RT (ms)	674.75				546.85			
SE	6.08				5.89			

### Visual ERP results

**Figure 2:**
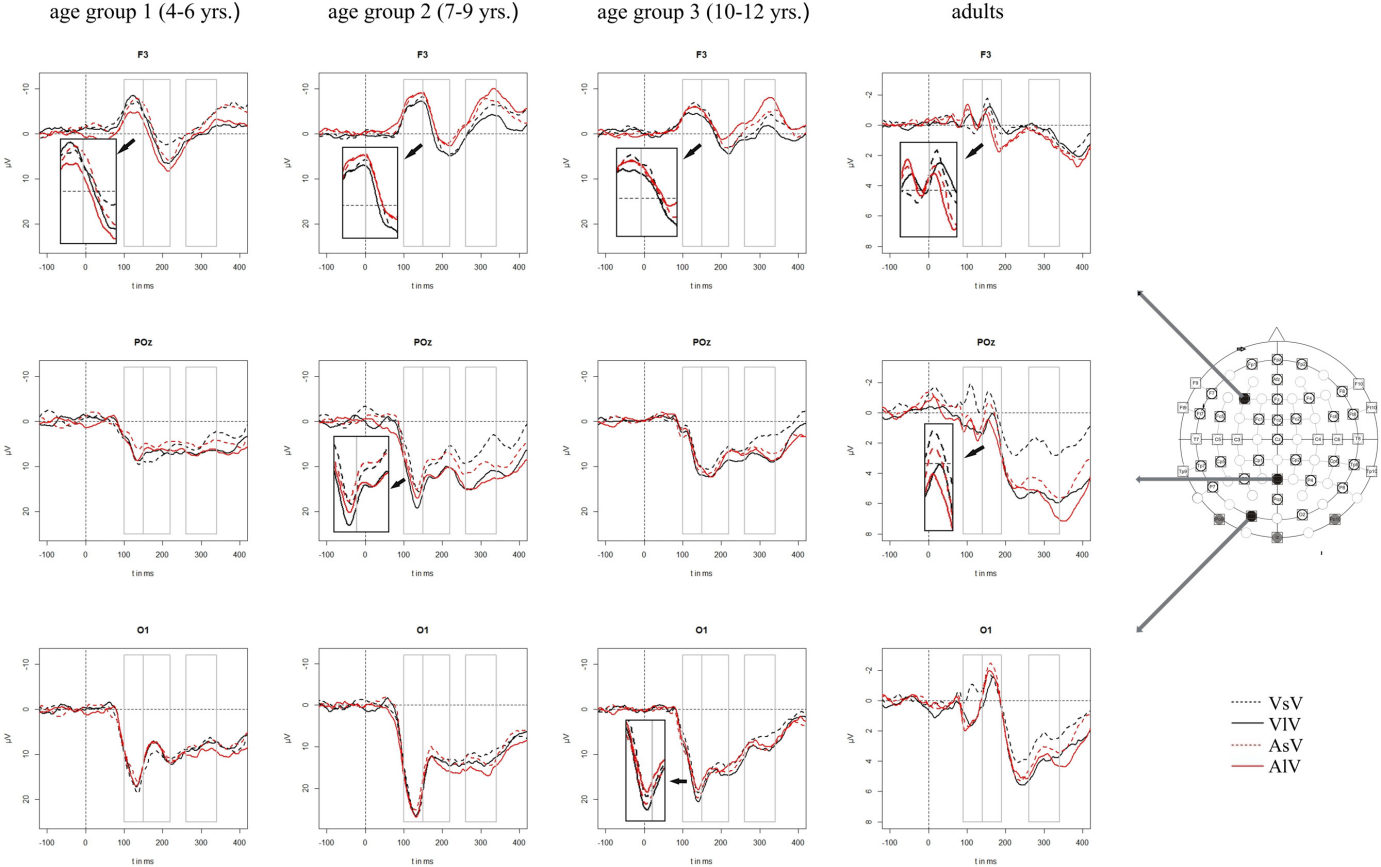
**Grand averaged ERPs to visual stimuli in the unimodal (black line) vs. crossmodal (red line) condition and after short (dashed line) vs. long (solid line) interstimulus interval**. ERPs are demonstrated at a subset of representative electrodes and separately for the four different age groups. Abbreviations: [vsv]: e.g., ERP to a visual stimulus preceded by a visual stimulus with a short ISI; a, auditory; v, visual; s, short ISI; l, long ISI. Time windows used for the statistical analyses are marked in gray. Negativity is plotted upwards.

In adults, visual stimuli elicited a positive potential between 100 and 110 ms post stimulus with a maximum at occipital electrode sites. The following negative peak reached its maximum around 160 ms post stimulus over occipital brain regions. In children, ERPs showed a positive potential at occipital electrodes with a maximum peak around 130–140 ms post stimulus. The following negative peak at parieto-occipital electrodes reached its maximum around 170–190 ms post stimulus (age group 1), 180–200 ms post stimulus (age group 2) and 210 ms post stimulus (age group 3), respectively. Concurrent with the positive peak over the occipital scalp, a negative potential was elicited over frontal and central brain regions with a maximum around 130 ms post stimulus followed by a positive deflection at 210–220 ms post stimulus. Figure 2 presents the grand averaged ERPs to visual stimuli at representative electrodes for (i) the four stimulus condition, i.e., unimodal vs. crossmodal condition and short ISI vs. long ISI, and (ii) the four age groups.

In all time windows, significant or marginally significant interactions of MT, ISI, one or more topographical factors and age group pointed toward ERP amplitude differences as a function of the preceding stimulus modality, the preceding ISI and the age of the participants [time window 100–150 ms (children)/90–140 ms (adults): ISI × MT × H × AP × Age group, *F*_(1, 52)_ = 6.03, *p* < 0.001]: ISI × MT × H × AP × E × Age group, *F*_(5, 260)_ = 2.37, *p* < 0.01, time window 150–220 ms (children)/140–190 ms (adults): ISI × MT × H × AP × Age group, *F*_(1, 52)_ = 2.52, *p* < 0.1, time window 260–340 ms (children/adults): ISI × MT × H × E × Age group, *F*_(5, 260)_ = 1.58, *p* < 0.1. Due to the interaction with the between-subject-factor Age group, the age groups were analyzed separately in the following analyses.

#### Unimodal refractory period effects

In adults and in age group 3 (10–12 years), ERP amplitudes in all analyzed time windows were affected by both the stimulus modality and the ISI and these effects depended on the recording sites (see significant main effect for ISI and/or for MT and/or interactions of MT × ISI × topographical factor, Tables 4, **7**). In the *post-hoc* analyses for age group 3 and adults, significant unimodal refractory period effects (vlv-vsv) were mainly found over the posterior scalp in all analyzed time windows (**Figure 4**). In age group 1 (4–6 years), significant interactions of MT, ISI and topographical factors in the Five-Way-ANOVA pointed toward amplitude differences between 100 and 150 ms as a function of the preceding stimulus modality, the preceding ISI and the recording site (see interactions of MT × ISI × topographical factor in Table 5): ERP amplitudes at occipital recording sites were larger in the unimodal condition when a short ISI compared to when a long ISI preceded. In contrast, over frontal and fronto-central regions amplitude deflections were larger in the unimodal condition when a long ISI compared to when a short ISI preceded (Figure 2). In the time windows 150–220 ms and 260–340 ms, significant or marginally significant main effects of ISI and significant interactions between ISI and topographical factors in all calculated ANOVAs reflected the influence of the preceding ISI rather than the preceding modality on the ERP amplitude (see main effect of ISI and interactions of ISI × topographical factor in Table 5). Unimodal refractory effects were mainly found at frontal and fronto-central recording sites (Figures 2, **4**). In age group 2 (7–9 years), significant or marginally significant main effect for ISI and significant interactions of ISI × one or more topographical factors in all analyzed time windows indicated unimodal refractory effects in this age group (see main effect of ISI and interactions of ISI × topographical factor in Table 6).

**Table 4A:** ***F*-values of the overall and subANOVAs (unimodal, crossmodal) for visual ERPs for the adult age-group, separately for each time window (90–140 ms, 140–190 ms, 260–340 ms)**.

**Factor (df1,df2^§^)**	**Visual**								
	**90–140 ms**			**140–190 ms**			**260–340 ms**		
	**Overall**	**Unimodal**	**Crossmodal**	**Overall**	**Unimodal**	**Crossmodal**	**Overall**	**Unimodal**	**Crossmodal**
ISI (1.14)	ns	ns	ns	ns	ns	ns	7.93^**^	10.3^**^	ns
ISI × AP (1.14)	7.53^*^	11.6^**^	ns	ns	3.9	ns	18.3^***^	16.3^***^	ns
ISI × E (5.70)	5.36^**^	5.89^**^	ns	ns	ns	ns	2.25	2.79^*^	ns
ISI × H (1.14)	ns	ns	ns	ns	ns	3.62	ns	ns	ns
ISI × AP × E (5.70)	2.32	4.68^**^	ns	ns	ns	ns	ns	ns	ns
ISI × AP × H (1.14)	ns	ns	ns	6.26^*^	7.2^*^	ns	9.48^**^	11.7^**^	ns
ISI × H × E (5.70)	ns	ns	ns	ns	ns	1.9	ns	ns	ns
ISI × AP × H × E (5.70)	ns	ns	Ns	ns	ns	ns	ns	ns	ns
MT (1.14)	ns	–	–	15.94^***^	–	–	9.15^**^	–	–
MT × ISI (1.14)	ns	–	–	ns	–	–	ns	–	–
MT × ISI × AP (1.14)	3.86	–	–	ns	–	–	3.88	–	–
MT × ISI × H (1.14)	ns	–	–	ns	–	–	ns	–	–
MT × ISI × E (5.70)	2.29	–	–	4.78^*^	–	–	ns	–	–
MT × ISI × AP × E (5.70)	2.92	–	–	ns	–	–	ns	–	–
MT × ISI × AP × H (1.14)	ns	–	–	ns	–	–	ns	–	–
MT × ISI × H × E (5.70)	ns	–	–	ns	–	–	ns	–	–
MT × ISI × AP × H × E (5.70)	2.28	–	–	ns	–	–	ns	–	–

Abbreviations: ISI, interstimulus interval; MT, Modality Transition; AP, Anterior Posterior; H, Hemisphere; E, Electrodes. P-value indicated by asterisk:

* p ≤ 0.05;

** p ≤ 0.01;

*** *p ≤ 0.001; no asterisk, trend, ns, non-significant. ANOVA type indicated by: overall = Five-Way ANOVA; unimodal/crossmodal = Four-Way ANOVA*.

§ *Uncorrected df-values but, if appropriate, Huynh-Feldt corrected p-values are reported*.

**Table 4B:** ***F*-values of the overall and subANOVAs (unimodal, crossmodal) for auditory ERPs for the adult age-group, separately for each time window (70–120 ms, 140–250 ms, 260–340 ms)**.

**Factor (df1,df2^§^)**	**Auditory**								
	**70–120 ms**			**140–250 ms**			**260–340 ms**		
	**Overall**	**Unimodal**	**Crossmodal**	**Overall**	**Unimodal**	**Crossmodal**	**Overall**	**Unimodal**	**Crossmodal**
ISI (1.14)	ns	ns	ns	8.38^*^	28.7^***^	ns	12.5^**^	14.9^**^	5.59^**^
ISI × AP (1.14)	ns	4.9^*^	ns	ns	ns	ns	25.7^***^	ns	ns
ISI × E (5.70)	3.68^*^	5.99^**^	ns	14.74	23.8^***^	3.1^*^	6.82^***^	5.43^**^	3.19^*^
ISI × H (1.14)	ns	ns	ns	ns	ns	ns	ns	ns	ns
ISI × AP × E (5.70)	9.82^***^	12.9^***^	ns	3.84^*^	3.84^*^	ns	ns	ns	ns
ISI × AP × H (1.14)	ns	ns	ns	ns	ns	ns	ns	ns	ns
ISI × H × E (5.70)	2.23	2.45^*^	ns	ns	ns	ns	ns	ns	ns
ISI × AP × H × E (5.70)	ns	2.31	ns	2.82^*^	4.56^**^	ns	2.74^*^	4.24^**^	ns
MT (1.14)	ns	–	–	59.9^***^	–	–	ns	–	–
MT × ISI (1.14)	ns	–	–	15.7^***^	–	–	ns	–	–
MT × ISI × AP (1.14)	ns	–	–	3.84	–	–	ns	–	–
MT × ISI × H (1.14)	ns	–	–	5.28^**^	–	–	ns	–	–
MT × ISI × E (5.70)	ns	–	–	ns	–	–	ns	–	–
MT × ISI × AP × E (5.70)	2.99	–	–	ns	–	–	ns	–	–
MT × ISI × AP × H (1.14)	ns	–	–	ns	–	–	ns	–	–
MT × ISI × H × E (5.70)	ns	–	–	ns	–	–	ns	–	–
MT × ISI × AP × H × E (5.70)	2.15	–	–	2.01	–	–	2.04	–	–

Abbreviations: ISI, interstimulus interval; MT, Modality Transition; AP, Anterior Posterior; H, Hemisphere; E, Electrodes. P-value indicated by asterisk:

* p ≤ 0.05;

** p ≤ 0.01;

*** *p ≤ 0.001; no asterisk, trend; ns, non-significant. ANOVA type indicated by: overall = Five-Way ANOVA; unimodal/crossmodal = Four-Way ANOVA*.

§ *Uncorrected df-values but, if appropriate, Huynh-Feldt corrected p-values are reported*.

**Table 4C:** ***F*-values of the overall and subANOVAs (unimodal, crossmodal) for visual and auditory ERPs at midline electrodes for the adult age-group, separately for each time window (visual: 90–140 ms, 140–190 ms, 260–340 ms, auditory: 70–120 ms, 140–250 ms, 260–340 ms)**.

**Factor (df1,df2^§^)**	**Visual**								
	**90–140 ms**			**140–190 ms**			**260–340 ms**		
	**Overall**	**Unimodal**	**Crossmodal**	**Overall**	**Unimodal**	**Crossmodal**	**Overall**	**Unimodal**	**Crossmodal**
ISI (1.14)	ns	ns	ns	ns	ns	ns	8.23^*^	8.74^**^	ns
ISI × E (5.70)	11.68^***^	16.8^***^	ns	6.27^**^	7.9^***^	ns	20.2^***^	33.1^***^	ns
MT (1.14)	ns	–	–	19.04^***^	–	–	8.16^*^	–	–
MT × ISI (1.14)	ns	–	–	ns	–	–	ns	–	–
MT × ISI × E (5.70)	7.48^***^	–	–	2.44	–	–	12.2^***^	–	–
**Factor (df1,df2^§^)**	**Auditory**								
	**70–120 ms**			**140–250 ms**			**260–340 ms**		
	**Overall**	**Unimodal**	**Crossmodal**	**Overall**	**Unimodal**	**Crossmodal**	**Overall**	**Unimodal**	**Crossmodal**
ISI (1.14)	ns	ns	ns	19.1^***^	60.4^***^	ns	17.0^***^	24.4^***^	7.43^**^
ISI × E (5.70)	9.81^**^	8.7^***^	5.66^**^	3.9^*^	2.27	9.1^***^	2.1	ns	ns
MT (1.14)	ns	–	–	74.2^***^	–	–	18.2^***^	–	–
MT × ISI (1.14)	ns	–	–	13.82^***^	–	–	ns	–	–
MT × ISI × E (5.70)	3.84^*^	–	–	6.1^**^	–	–	ns	–	–

Abbreviations: ISI, interstimulus interval; MT, Modality Transition; AP, Anterior Posterior; H, Hemisphere; E, Electrodes. P-value indicated by asterisk:

* p ≤ 0.05;

** p ≤ 0.01;

*** *p ≤ 0.001; no asterisk, trend; ns, non-significant. ANOVA type indicated by: overall = Three-Way ANOVA; unimodal/crossmodal = Two-Way ANOVA*.

§ *Uncorrected df-values but, if appropriate, Huynh-Feldt corrected p-values are reported*.

**Table 5A:** ***F*-values of the overall and subANOVAs (unimodal, crossmodal) for visual ERPs for age-group 1 (4–6 years), separately for each time window (100–150 ms, 150–220 ms, 260–340 ms)**.

**Factor (df1,df2^§^)**	**Visual**								
	**100–150 ms**			**150–220 ms**			**260–340 ms**		
	**Overall**	**Unimodal**	**Crossmodal**	**Overall**	**Unimodal**	**Crossmodal**	**Overall**	**Unimodal**	**Crossmodal**
ISI (1.14)	ns	ns	ns	4.3	ns	ns	ns	3.46	ns
ISI × AP (1.14)	ns	ns	ns	8.6^**^	3.69	4.64	ns	ns	ns
ISI × E (5.70)	ns	ns	ns	2.34	ns	2.19	2.78^*^	ns	ns
ISI × H (1.14)	ns	ns	ns	3.53	ns	ns	ns	ns	ns
ISI × AP × E (5.70)	ns	ns	ns	ns	ns	ns	ns	3.08	ns
ISI × AP × H (1.14)	ns	5.45^*^	4.73^*^	5.34^*^	4.8^*^	ns	4.2	3.48	ns
ISI × H × E (5.70)	ns	ns	ns	ns	ns	ns	ns	ns	ns
ISI × AP × H × E (5.70)	ns	3.44^**^	ns	2.29	1.9	ns	2.36	3.05	ns
MT (1.14)	ns	–	–	ns	–	–	ns	–	–
MT × ISI (1.14)	ns	–	–	ns	–	–	ns	–	–
MT × ISI × AP (1.14)	ns	–	–	ns	–	–	ns	–	–
MT × ISI × H (1.14)	ns	–	–	ns	–	–	ns	–	–
MT × ISI × E (5.70)	ns	–	–	ns	–	–	ns	–	–
MT × ISI × AP × E (5.70)	ns	–	–	ns	–	–	ns	–	–
MT × ISI × AP × H (1.14)	7.88^*^	–	–	ns	–	–	ns	–	–
MT × ISI × H × E (5.70)	ns	–	–	ns	–	–	2.04	–	–
MT × ISI × AP × H × E (5.70)	2.87^*^	–	–	ns	–	–	ns	–	–

Abbreviations: ISI, interstimulus interval; MT, Modality Transition; AP, Anterior Posterior; H, Hemisphere; E, Electrodes. P-value indicated by asterisk:

* p ≤ 0.05;

** p ≤ 0.01;

^***^p ≤ 0.001; no asterisk, trend; ns, non-significant. ANOVA type indicated by: overall = Five-Way ANOVA; unimodal/crossmodal = Four-Way ANOVA.

§ *Uncorrected df-values but, if appropriate, Huynh-Feldt corrected p-values are reported*.

**Table 5B:** ***F*-values of the overall and subANOVAs (unimodal, crossmodal) for auditory ERPs for age–group 1 (4–6 years), separately for each time window (90–150 ms, 150–250 ms, 260–340 ms)**.

**Factor (df1,df2^§^)**	**Auditory**								
	**90–150 ms**			**150–250 ms**			**260–340 ms**		
	**Overall**	**Unimodal**	**Crossmodal**	**Overall**	**Unimodal**	**Crossmodal**	**Overall**	**Unimodal**	**Crossmodal**
ISI (1.14)	ns	ns	ns	7.18^**^	9.34^**^	3.36	9.97^**^	7.57^*^	4.13
ISI × AP (1.14)	12.08^**^	4.16	14.0^**^	17.7^***^	9.54^**^	9.75^**^	5.62^*^	ns	3.45
ISI × E (5.70)	2.55^*^	3.25^*^	ns	11.0^***^	10.7^***^	4.98^**^	9.52^***^	7.1^***^	4.16^*^
ISI × H (1.14)	ns	ns	ns	ns	ns	ns	ns	4.47	ns
ISI × AP × E (5.70)	ns	ns	ns	ns	2.29	ns	ns	ns	ns
ISI × AP × H (1.14)	3.46	ns	ns	5.04^*^	ns	3.18	15.82^*^	3.63	6.27^*^
ISI × H × E (5.70)	ns	ns	ns	ns	ns	ns	ns	ns	ns
ISI × AP × H × E (5.70)	ns	ns	ns	ns	ns	ns	ns	ns	ns
MT (1.14)	ns	–	–	ns	–	–	10.47^**^	–	–
MT × ISI (1.14)	ns	–	–	ns	–	–	ns	–	–
MT × ISI × AP (1.14)	ns	–	–	ns	–	–	ns	–	–
MT × ISI × H (1.14)	ns	–	–	ns	–	–	ns	–	–
MT × ISI × E (5.70)	2.04	–	–	ns	–	–	ns	–	–
MT × ISI × AP × E (5.70)	ns	–	–	ns	–	–	ns	–	–
MT × ISI × AP × H (1.14)	ns	–	–	ns	–	–	ns	–	–
MT × ISI × H × E (5.70)	ns	–	–	ns	–	–	ns	–	–
MT × ISI × AP × H × E (5.70)	ns	–	–	ns	–	–	ns	–	–

Abbreviations: ISI, interstimulus interval; MT, Modality Transition; AP, Anterior Posterior; H, Hemisphere; E, Electrodes. P-value indicated by asterisk:

* p ≤ 0.05;

** p ≤ 0.01;

*** *p ≤ 0.001; no asterisk, trend; ns, non-significant. ANOVA type indicated by: overall = Five-Way ANOVA; unimodal/crossmodal = Four-Way ANOVA*.

§ *Uncorrected df-values but, if appropriate, Huynh-Feldt corrected p-values are reported*.

**Table 5C:** ***F*-values of the overall and subANOVAs (unimodal, crossmodal) for visual and auditory ERPs at midline electrodes for age-group 1 (4–6 years), separately for each time window (visual: 100–150 ms, 150–220 ms, 260–340 ms, auditory: 90–150 ms, 150–250 ms, 260–340 ms)**.

**Factor (df1,df2^§^)**	**Visual**								
	**100–150 ms**			**150–220 ms**			**260–340 ms**		
	**Overall**	**Unimodal**	**Crossmodal**	**Overall**	**Unimodal**	**Crossmodal**	**Overall**	**Unimodal**	**Crossmodal**
ISI (1.14)	ns	ns	ns	8.7^***^	2.32	4.05	ns	6.07,^*^	ns
ISI × E (6.84)	ns	ns	ns	ns	ns	ns	ns	4.32^**^	ns
MT (1.14)	ns	–	–	ns	–	–	ns	–	–
MT × ISI (1.14)	ns	–	–	ns	–	–	ns	–	–
MT × ISI × E (6.84)	ns	–	–	ns	–	–	ns	–	–
**Factor (df1,df2^§^)**	**Auditory**								
	**90–150 ms**			**150–250 ms**			**260–340 ms**		
	**Overall**	**Unimodal**	**Crossmodal**	**Overall**	**Unimodal**	**Crossmodal**	**Overall**	**Unimodal**	**Crossmodal**
ISI (1.14)	4.47	4.02	ns	13.0^**^	22.4^***^	4.76^*^	11.99^**^	11.83^**^	4.09
ISI × E (6.84)	ns	ns	3.55^*^	ns	ns	ns	ns	ns	ns
MT (1.14)	ns	–	–	ns	–	–	16.8^***^	–	–
MT × ISI (1.14)	ns	–	–	ns	–	–	ns	–	–
MT × ISI × E (6.84)	2.69^*^	–	–	ns	–	–	ns	–	–

Abbreviations: ISI, interstimulus interval; MT, Modality Transition; AP, Anterior Posterior; H, Hemisphere; E, Electrodes. P-value indicated by asterisk:

* p ≤ 0.05;

** p ≤ 0.01;

*** *p ≤ 0.001; no asterisk, trend; ns, non-significant. ANOVA type indicated by: overall = Three-Way ANOVA; unimodal/crossmodal = Two-Way ANOVA*.

§ *Uncorrected df-values but, if appropriate, Huynh-Feldt corrected p-values are reported*.

**Table 6A:** ***F*-values of the overall and subANOVAs (unimodal, crossmodal) for visual ERPs for age-group 2 (7–9 years), separately for each time window (100–150 ms, 150–220 ms, 260–340 ms)**.

**Factor (df1,df2^§^)**	**Visual**								
	**100–150 ms**			**150–220 ms**			**260–340 ms**		
	**Overall**	**Unimodal**	**Crossmodal**	**Overall**	**Unimodal**	**Crossmodal**	**Overall**	**Unimodal**	**Crossmodal**
ISI (1.12)	5.59^*^	3.44	ns	6.41^*^	3.93	3.41	4.57	3.56	ns
ISI × AP (1.12)	ns	ns	ns	ns	ns	3.92	4.88	ns	ns
ISI × E (5.60)	ns	ns	ns	ns	ns	ns	2.93^*^	2.49^*^	ns
ISI × H (1.12)	ns	ns	ns	3.46	ns	6.48^*^	ns	ns	ns
ISI × AP × E (5.60)	ns	ns	ns	ns	ns	ns	ns	ns	ns
ISI × AP × H (1.12)	ns	ns	ns	ns	ns	ns	ns	ns	ns
ISI × H × E (5.60)	ns	ns	ns	ns	ns	ns	ns	ns	ns
ISI × AP × H × E (5.60)	ns	ns	ns	ns	ns	ns	ns	ns	ns
MT (1.12)	ns	–	–	ns	–	–	ns	–	–
MT × ISI (1.12)	ns	–	–	ns	–	–	ns	–	–
MT × ISI × AP (1.12)	ns	–	–	ns	–	–	ns	–	–
MT × ISI × H (1.12)	ns	–	–	ns	–	–	ns	–	–
MT × ISI × E (5.60)	ns	–	–	ns	–	–	ns	–	–
MT × ISI × AP × E (5.60)	ns	–	–	ns	–	–	ns	–	–
MT × ISI × AP × H (1.12)	ns	–	–	ns	–	–	ns	–	–
MT × ISI × H × E (5.60)	ns	–	–	ns	–	–	ns	–	–
MT × ISI × AP × H × E (5.60)	ns	–	–	ns	–	–	ns	–	–

Abbreviations: ISI, interstimulus interval; MT, Modality Transition; AP, Anterior Posterior; H, Hemisphere; E, Electrodes. P-value indicated by asterisk:

* p ≤ 0.05;

^**^p ≤ 0.01; ^***^p ≤ 0.001; no asterisk, trend; ns, non-significant. ANOVA type indicated by: overall = Five-Way ANOVA; unimodal/crossmodal = Four-Way ANOVA.

§ *Uncorrected df-values but, if appropriate, Huynh-Feldt corrected p-values are reported*.

**Table 6B:** ***F*-values of the overall and subANOVAs (unimodal, crossmodal) for auditory ERPs for age-group 2 (7–9 years), separately for each time window (90–150 ms, 150–250 ms, 260–340 ms)**.

**Factor (df1,df2^§^)**	**Auditory**								
	**90–150 ms**			**150–250 ms**			**260–340 ms**		
	**Overall**	**Unimodal**	**Crossmodal**	**Overall**	**Unimodal**	**Crossmodal**	**Overall**	**Unimodal**	**Crossmodal**
ISI (1.12)	ns	ns	ns	ns	5.94^*^	ns	ns	3.36	ns
ISI × AP (1.12)	ns	ns	ns	ns	ns	ns	ns	ns	ns
ISI × E (5.60)	2.69^*^	ns	ns	8.95^***^	11.9^***^	ns	ns	3.78^*^	ns
ISI × H (1.12)	ns	ns	ns	ns	ns	ns	ns	ns	ns
ISI × AP × E (5.60)	2.23	ns	ns	6.21^***^	5.8^***^	ns	ns	ns	ns
ISI × AP × H (1.12)	ns	ns	ns	ns	ns	ns	ns	ns	ns
ISI × H × E (5.60)	ns	ns	ns	ns	ns	ns	ns	ns	ns
ISI × AP × H × E (5.60)	ns	ns	ns	ns	ns	ns	ns	ns	ns
MT (1.12)	ns	–	–	15.95^**^	–	–	8.25^*^	–	–
MT × ISI (1.12)	ns	–	–	5.25^*^	–	–	ns	–	–
MT × ISI × AP (1.12)	ns	–	–	ns	–	–	ns	–	–
MT × ISI × H (1.12)	ns	–	–	ns	–	–	ns	–	–
MT × ISI × E (5.60)	ns	–	–	5.95^***^	–	–	2.92^*^	–	–
MT × ISI × AP × E (5.60)	ns	–	–	ns	–	–	ns	–	–
MT × ISI × AP × H (1.12)	ns	–	–	ns	–	–	ns	–	–
MT × ISI × H × E (5.60)	ns	–	–	ns	–	–	ns	–	–
MT × ISI × AP × H × E (5.60)	ns	–	–	ns	–	–	ns	–	–

Abbreviations: ISI, interstimulus interval; MT, Modality Transition; AP, Anterior Posterior; H, Hemisphere; E, Electrodes. P-value indicated by asterisk:

* p ≤ 0.05;

** p ≤ 0.01;

*** *p ≤ 0.001; no asterisk, trend; ns, non-significant. ANOVA type indicated by: overall = Five-Way ANOVA; unimodal/crossmodal = Four-Way ANOVA*.

§ *Uncorrected df-values but, if appropriate, Huynh-Feldt corrected p-values are reported*.

**Table 6C:** ***F*-values of the overall and subANOVAs (unimodal, crossmodal) for visual and auditory ERPs at midline electrodes for age-group 2 (7–9 years), separately for each time window (visual: 100–150 ms, 150–220 ms, 260–340 ms, auditory: 90–150 ms, 150–250 ms, 260–340 ms)**.

**Factor (df1,df2^§^)**	**Visual**								
	**100–150 ms**			**150–220 ms**			**260–340 ms**		
	**Overall**	**Unimodal**	**Crossmodal**	**Overall**	**Unimodal**	**Crossmodal**	**Overall**	**Unimodal**	**Crossmodal**
ISI (1.12)	3.87	ns	ns	4.12	ns	ns	3.83	6.16^*^	ns
ISI × E (6.72)	5.31^**^	5.3^**^	ns	4.47^*^	ns	3.67^*^	7.02^**^	3.98^*^	3.73^*^
MT (1.12)	ns	–	–	ns	–	–	ns	–	–
MT × ISI (1.12)	ns	–	–	ns	–	–	ns	–	–
MT × ISI × E (6.72)	ns	–	–	ns	–	–	ns	–	–
**Factor (df1,df2^§^)**	**Auditory**								
	**90–150 ms**			**150–250 ms**			**260–340 ms**		
	**Overall**	**Unimodal**	**Crossmodal**	**Overall**	**Unimodal**	**Crossmodal**	**Overall**	**Unimodal**	**Crossmodal**
ISI (1.12)	3.27	ns	4.17	6.89^*^	10.34^**^	ns	ns	ns	ns
ISI × E (6.72)	ns	ns	ns	2.98^*^	3.53^*^	ns	ns	ns	2.2
MT (1.12)	ns	–	–	15.35^**^	–	–	3.48	–	–
MT × ISI (1.12)	ns	–	–	8.54^**^	–	–	ns	–	–
MT × ISI × E (6.72)	ns	–	–	2.28	–	–	ns	–	–

Abbreviations: ISI, interstimulus interval; MT, Modality Transition; AP, Anterior Posterior; H, Hemisphere; E, Electrodes. P-value indicated by asterisk:

* p ≤ 0.05;

** p ≤ 0.01;

^***^p ≤ 0.001; no asterisk, trend; ns, non-significant. ANOVA type indicated by: overall = Three-Way ANOVA; unimodal/crossmodal = Two-Way ANOVA.

§ *Uncorrected df-values but, if appropriate, Huynh-Feldt corrected p-values are reported*.

#### Crossmodal refractory period effects

In the youngest age group (4–6 years), ERP amplitudes in the time window 100–150 ms were modified by the preceding modality, the preceding ISI and the recording site (see interactions of MT × ISI × topographical factors in Table 5) and significant crossmodal refractory period effects (alv-asv) were observed at fronto-central and fronto-temporal recording sites in the *post-hoc* analyses (**Figure 4**). In adults and age group 3 (10–12 years), significant interactions of MT, ISI and topographical factors in the Five-Way- and Three-Way-ANOVA indicated that amplitude differences were affected by the preceding stimulus modality and the ISI. These effects depended on the recording site (see interactions of MT × ISI × topographical factor in Tables 4 and 7). In contrast to the results in age group 1, crossmodal refractory effects (alv-asv) were confirmed at single parieto-occipital and occipital electrodes, respectively (Figures 2, 4), and after 150 ms post stimulus. In age group 2 (7–9 years), the preceding ISI and the recording site rather than the MT influenced ERP amplitudes (see main effect of ISI and interactions of ISI × topographical factor in Table 6).

**Table 7A:** ***F*-values of the overall and subANOVAs (unimodal, crossmodal) for visual ERPs for age-group 3 (10–12 years), separately for each time window (100–150 ms, 150–220 ms, 260–340 ms)**.

**Factor (df1,df2^§^)**	**Visual**								
	**100–150 ms**			**150–220 ms**			**260–340 ms**		
	**Overall**	**Unimodal**	**Crossmodal**	**Overall**	**Unimodal**	**Crossmodal**	**Overall**	**Unimodal**	**Crossmodal**
ISI (1.12)	3.9	9.3^**^	ns	ns	5.92^*^	ns	ns	8.44^**^	ns
ISI × AP (1.12)	ns	ns	ns	ns	ns	ns	ns	ns	ns
ISI × E (5.60)	ns	ns	ns	ns	ns	ns	ns	2.61	ns
ISI × H (1.12)	ns	ns	3.45	ns	ns	ns	ns	ns	ns
ISI × AP × E (5.60)	ns	ns	ns	ns	2.33	ns	ns	2.95^*^	ns
ISI × AP × H (1.12)	ns	ns	ns	ns	ns	ns	ns	9.59^*^	ns
ISI × H × E (5.60)	ns	ns	ns	ns	ns	ns	ns	ns	ns
ISI × AP × H × E (5.60)	2.52^*^	ns	5.5^***^	ns	ns	ns	ns	ns	ns
MT (1.12)	ns	–	–	ns	–	–	5.94^*^	–	–
MT × ISI (1.12)	4.31	–	–	ns	–	–	3.54	–	–
MT × ISI × AP (1.12)	ns	–	–	ns	–	–	ns	–	–
MT × ISI × H (1.12)	7.33^*^	–	–	5.97^*^	–	–	ns	–	–
MT × ISI × E (5.60)	ns	–	–	ns	–	–	ns	–	–
MT × ISI × AP × E (5.60)	3.17^*^	–	–	2.39	–	–	4.61^**^	–	–
MT × ISI × AP × H (1.12)	ns	–	–	ns	–	–	ns	–	–
MT × ISI × H × E (5.60)	ns	–	–	ns	–	–	ns	–	–
MT × ISI × AP × H × E (5.60)	3.29^*^	–	–	ns	–	–	ns	–	–

Abbreviations: ISI, interstimulus interval; MT, Modality Transition; AP, Anterior Posterior; H, Hemisphere; E, Electrodes. P-value indicated by asterisk:

* p ≤ 0.05;

** p ≤ 0.01;

*** *p ≤ 0.001; no asterisk, trend; ns, non-significant. ANOVA type indicated by: overall = Five-Way ANOVA; unimodal/crossmodal = Four-Way ANOVA*.

§ *Uncorrected df-values but, if appropriate, Huynh-Feldt corrected p-values are reported*.

**Table 7B:** ***F*-values of the overall and subANOVAs (unimodal, crossmodal) for auditory ERPs for age-group 3 (10–12 years), separately for each time window (90–150 ms, 150–250 ms, 260–340 ms)**.

**Factor (df1,df2^§^)**	**Auditory**								
	**90–150 ms**			**150–250 ms**			**260–340 ms**		
	**Overall**	**Unimodal**	**Crossmodal**	**Overall**	**Unimodal**	**Crossmodal**	**Overall**	**Unimodal**	**Crossmodal**
ISI (1.12)	3.89	3.26	ns	15.39^**^	32.1^***^	ns	13.66^**^	9.73^**^	ns
ISI × AP (1.12)	ns	ns	ns	ns	ns	ns	ns	ns	ns
ISI × E (5.60)	2.96	ns	ns	8.03^***^	13.5^***^	ns	ns	2.22	ns
ISI × H (1.12)	ns	5.31^*^	ns	ns	ns	ns	ns	ns	ns
ISI × AP × E (5.60)	ns	ns	ns	2.27	3.25	ns	2.29	ns	ns
ISI × AP × H (1.12)	ns	ns	ns	ns	ns	ns	ns	ns	ns
ISI × H × E (5.60)	ns	ns	ns	ns	ns	3.54	ns	ns	ns
ISI × AP × H × E (5.60)	ns	ns	2.94^*^	2.14	ns	3.12^*^	2.89^*^	ns	3.27^*^
MT (1.12)	ns	–	–	ns	–	–	ns	–	–
MT × ISI (1.12)	ns	–	–	3.28	–	–	ns	–	–
MT × ISI × AP (1.12)	ns	–	–	ns	–	–	ns	–	–
MT × ISI × H (1.12)	ns	–	–	ns	–	–	ns	–	–
MT × ISI × E (5.60)	ns	–	–	2.76	–	–	ns	–	–
MT × ISI × AP × E (5.60)	ns	–	–	ns	–	–	ns	–	–
MT × ISI × AP × H (1.12)	ns	–	–	ns	–	–	ns	–	–
MT × ISI × H × E (5.60)	ns	–	–	ns	–	–	ns	–	–
MT × ISI × AP × H × E (5.60)	ns	–	–	ns	–	–	ns	–	–

Abbreviations: ISI, interstimulus interval; MT, Modality Transition; AP, Anterior Posterior; H, Hemisphere; E, Electrodes. P-value indicated by asterisk:

* p ≤ 0.05;

** p ≤ 0.01;

*** *p ≤ 0.001; no asterisk, trend; ns, non-significant. ANOVA type indicated by: overall = Five-Way ANOVA; unimodal/crossmodal = Four-Way ANOVA*.

§ *Uncorrected df-values but, if appropriate, Huynh-Feldt corrected p-values are reported*.

**Table 7C:** ***F*-values of the overall and subANOVAs (unimoda, crossmodal) for visual and auditory ERPs at midline electrodes for age-group 3 (10–12 years), separately for each time window (visual: 100–150 ms, 150–220 ms, 260–340 ms, auditory: 90–150 ms, 150–250 ms, 260–340 ms)**.

**Factor (df1,df2^§^)**	**Visual**								
	**100–150 ms**			**150–220 ms**			**260–340 ms**		
	**Overall**	**Unimodal**	**Crossmodal**	**Overall**	**Unimodal**	**Crossmodal**	**Overall**	**Unimodal**	**Crossmodal**
ISI (1.12)	ns	3.21	ns	ns	4.35	ns	ns	4.82^*^	ns
ISI × E (6.72)	ns	ns	ns	2.76	ns	ns	8.02^***^	4.06^*^	3.97^*^
MT (1.12)	ns	–	–	ns	–	–	3.75	–	–
MT × ISI (1.12)	ns	–	–	ns	–	–	ns	–	–
MT × ISI × E (6.72)	ns	–	–	ns	–	–	ns	–	–
**Factor (df1,df2^§^)**	**Auditory**								
	**90–150 ms**			**150–250 ms**			**260–340 ms**		
	**Overall**	**Unimodal**	**Crossmodal**	**Overall**	**Unimodal**	**Crossmodal**	**Overall**	**Unimodal**	**Crossmodal**
ISI (1.12)	ns	ns	ns	12.8^**^	43.1^***^	ns	6.37^*^	6.59^*^	ns
ISI × E (6.72)	ns	ns	ns	3.24^*^	ns	ns	ns	ns	ns
MT (1.12)	ns	–	–	ns	–	–	ns	–	–
MT × ISI (1.12)	ns	–	–	3.83	–	–	ns	–	–
MT × ISI × E (6.72)	ns	–	–	ns	–	–	ns	–	–

Abbreviations: ISI, interstimulus interval; MT, Modality Transition; AP, Anterior Posterior; H, Hemisphere; E, Electrodes. P-value indicated by asterisk:

* p ≤ 0.05;

** p ≤ 0.01;

*** *p ≤ 0.001; no asterisk, trend; ns, non-significant. ANOVA type indicated by: overall = Three-Way ANOVA; unimodal/crossmodal = Two-Way ANOVA*.

§ *Uncorrected df-values but, if appropriate, Huynh-Feldt corrected p-values are reported*.

### Auditory ERP results

**Figure 3:**
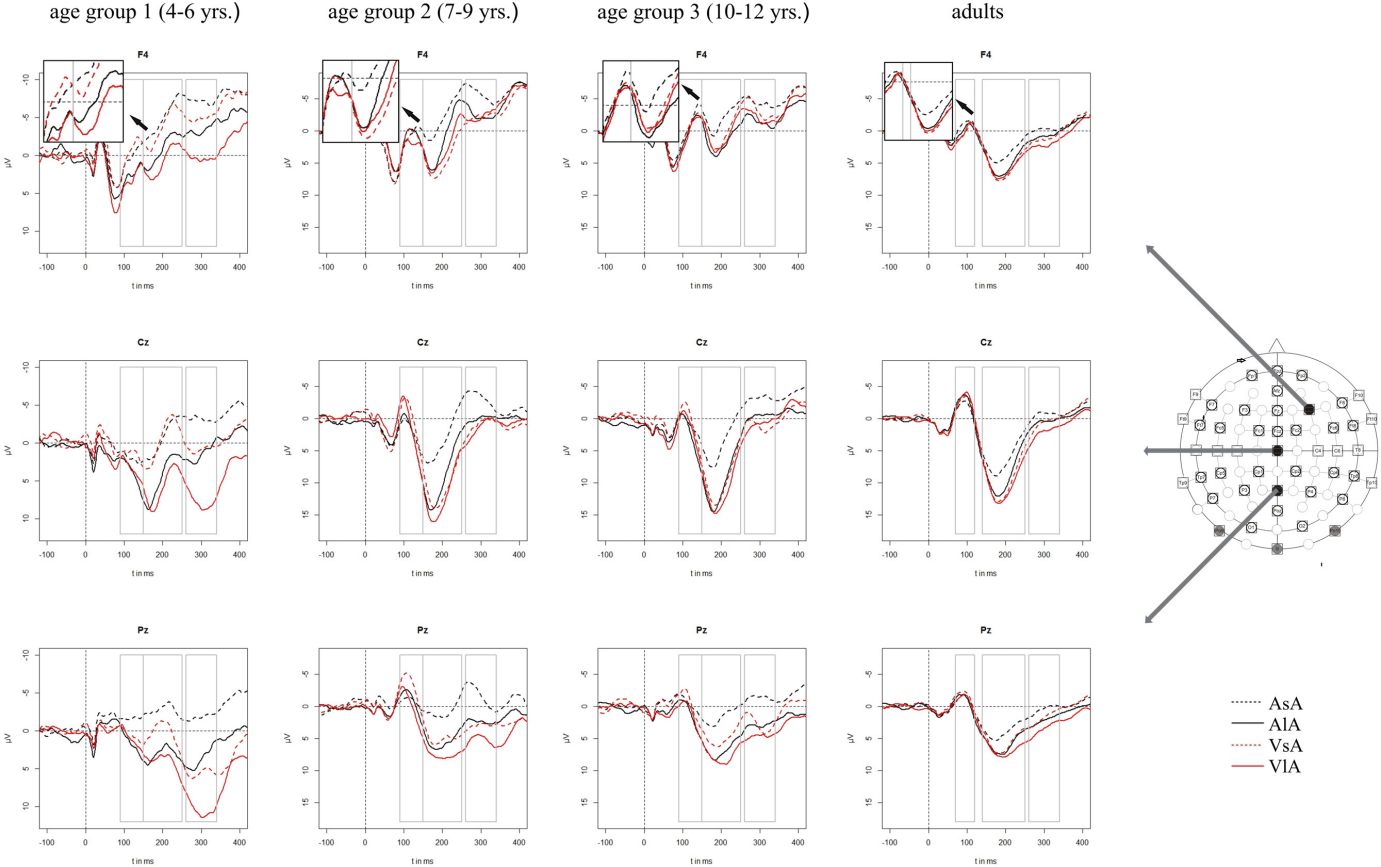
**Grand averaged ERPs to auditory stimuli in the unimodal (black line) vs. crossmodal (red line) condition and after short (dashed line) vs. long (solid line) interstimulus intervals**. ERPs are demonstrated at a subset of representative electrodes and for the four different age groups. Abbreviations: [asa]: e.g., ERP to an auditory stimulus preceded by an auditory stimulus with a short ISI; a, auditory; v, visual; s, short ISI; l, long ISI. Time windows used for the statistical analyses are marked in gray. Negativity is plotted upwards.

Auditory ERPs were characterized by a fronto-centrally distributed positive peak, a second, negative peak with a maximal deflection over the vertex and a third, positive peak with a vertex maximum as well. This ERP pattern was found in all age groups. The latencies of the first and the second peak decreased with increasing age: The first positive wave peaked around 60 ms post stimulus in adults and around 70 ms post stimulus in children. The next largest peak (with negative polarity) was between 80–90 ms post stimulus in adults and around 100 ms post stimulus in children. The third, positive peak showed a similar latency with approximately 180 ms post stimulus across the age groups. Figure 3 depicts the grand averaged ERPs to auditory stimuli at representative electrodes for (i) the four stimulus condition, i.e., unimodal vs. crossmodal condition and short ISI vs. long ISI, and (ii) the four age groups.

**Figure 4:**
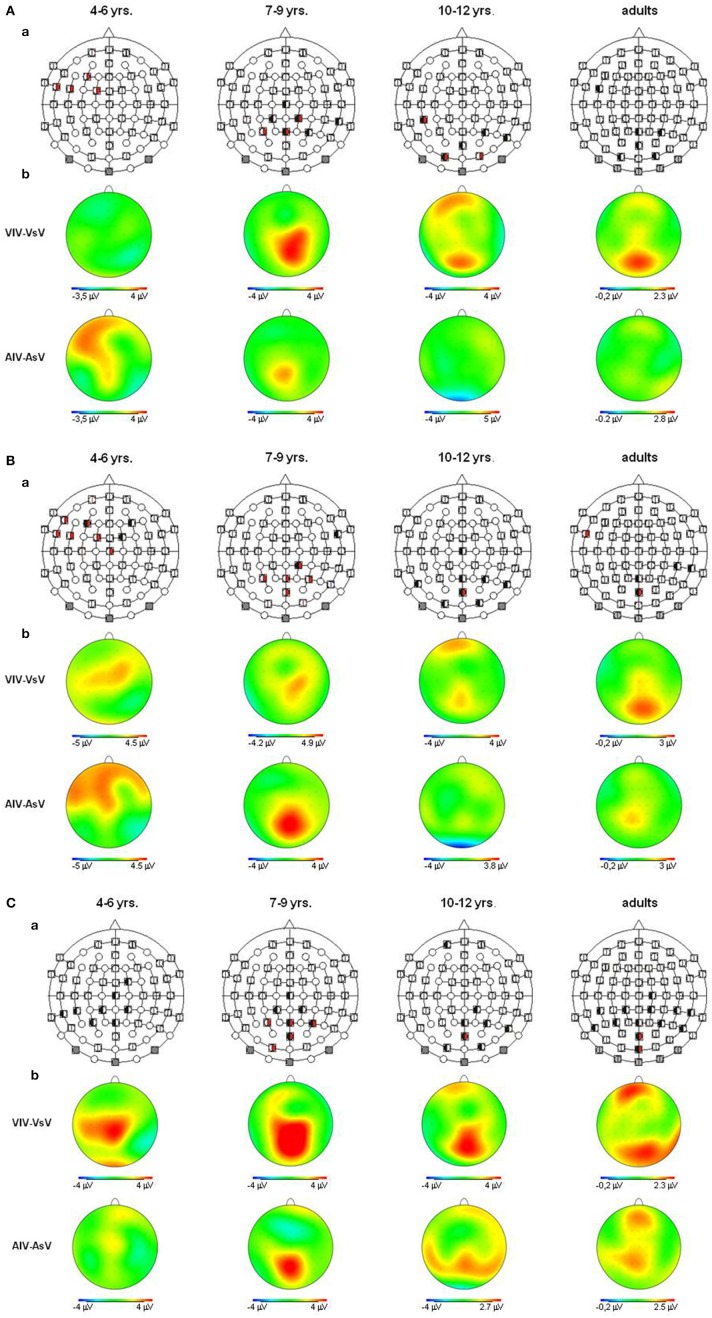
**(A)** Visual refractory period effects (*p* ≤ 0.05) in the unimodal (black, left segment) and the crossmodal (red, right segment) condition, separately illustrated for each age group **(a)**. Voltage difference maps of conditions with a long ISI minus conditions with a short ISI (unimodal vs. crossmodal) **(b)**. Analyzed time windows: 100–150 ms (children), 90–140 ms (adults). Abbreviations: [vsv]: e.g., ERP to a visual stimulus preceded by a visual stimulus with a short ISI: a, auditory; v, visual; s, short ISI; l, long ISI. **(B)** Visual refractory period effects (*p* ≤ 0.05) in the unimodal (black, left segment) and the crossmodal (red, right segment) condition, separately illustrated for each age group **(a)**. Voltage difference maps of conditions with a long ISI minus conditions with a short ISI (unimodal vs. crossmodal) **(b)**. Analyzed time windows: 150–220 ms (children), 140–190 ms (adults). Abbreviations: [vsv]: e.g., ERP to a visual stimulus preceded by a visual stimulus with a short ISI: a, auditory; v, visual; s, short ISI; l, long ISI. **(C)** Visual refractory period effects (*p* ≤ 0.05) in the unimodal (black, left segment) and the crossmodal (red, right segment) condition, separately illustrated for each age group **(a)**. Voltage difference maps of conditions with a long ISI minus conditions with a short ISI (unimodal vs. crossmodal) **(b)**. Analyzed time window: 260–340 ms in children and adults. Abbreviations: [vsv]: e.g., ERP to a visual stimulus preceded by a visual stimulus with a short ISI: a, auditory; v, visual; s, short ISI; l, long ISI.

In the first 250 ms post stimulus, ERPs to auditory stimuli showed refractory effects which depended on the preceding modality, the recording site and the age of the participant [time window 90–150 ms (children)/70–120 ms (adults): ISI × MT × AP × E × Age group, *F*_(5, 190)_ = 7.43, *p* < 0.001, time window 150–250 ms (children)/140–250 ms (adults): ISI × MT × E × Age group, *F*_(5, 260)_ = 2.065, *p* < 0.05]. Due to the interaction with the between-subject-factor Age group, the age groups were analyzed separately in the following analyses.

#### Unimodal refractory period effects

**Figure 5:**
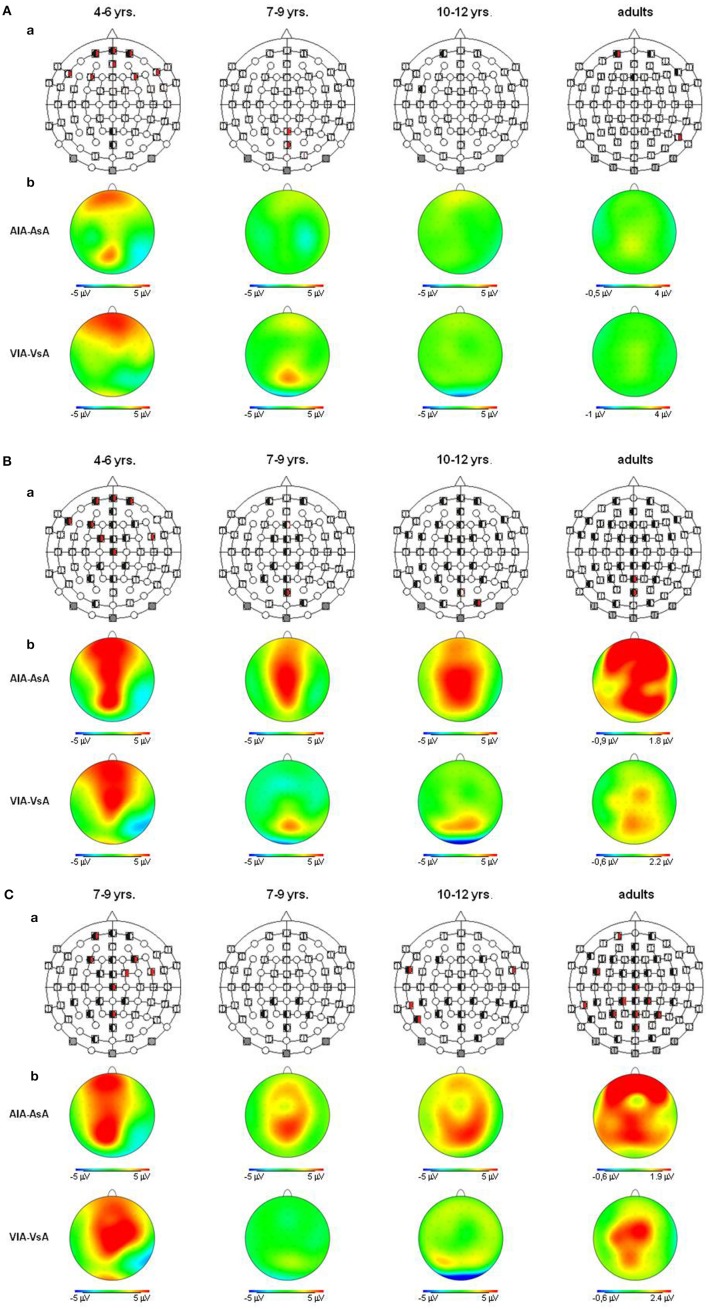
**(A)** Auditory refractory period effects (*p* ≤ 0.05) in the unimodal (black, left segment) and the crossmodal (red, right segment) condition, separately illustrated for each age group **(a)**. Voltage difference maps of conditions with a long ISI minus conditions with a short ISI (unimodal vs. crossmodal) **(b)**. Analyzed time windows: 90–150 ms (children), 70–120 ms (adults). Abbreviations: [asa]: e.g., ERP to an auditory stimulus preceded by a auditory stimulus with a short ISI: a, auditory; v, visual; s, short ISI; l, long ISI. **(B)** Auditory refractory period effects (*p* ≤ 0.05) in the unimodal (black, left segment) and the crossmodal (red, right segment) condition, separately illustrated for each age group **(a)**. Voltage difference maps of conditions with a long ISI minus conditions with a short ISI (unimodal vs. crossmodal) **(b)**. Analyzed time windows: 150–250 ms (children), 140–250 ms (adults). Abbreviations: [asa]: e.g., ERP to an auditory stimulus preceded by a auditory stimulus with a short ISI: a, auditory; v, visual; s, short ISI; l, long ISI. **(C)** Auditory refractory period effects (*p* ≤ 0.05) in the unimodal (black, left segment) and the crossmodal (red, right segment) condition, separately illustrated for each age group **(a)**. Voltage difference maps of conditions with a long ISI minus conditions with a short ISI (unimodal vs. crossmodal) **(b)**. Analyzed time window: 260–340 ms in both children and adults. Abbreviations: [asa]: e.g., ERP to an auditory stimulus preceded by a auditory stimulus with a short ISI: a, auditory; v, visual; s, short ISI; l, long ISI.

In all age groups, significant or marginally significant main effects of MT and ISI and/or interactions between MT, ISI and topographical factors (see Five-Way- and Three-Way ANOVA in Tables 4–7) reflected the influence of the preceding stimulus modality, the preceding ISI and the recording site on ERP amplitudes. In the time window 150–250 ms (children) and 140–250 ms (adults), respectively, significant unimodal refractory effects (ala-asa) were confirmed along the midline electrodes in age group 1 (4–6 years) and age group 2 (7–9 years), and widespread over the scalp in the oldest children and in adults. In age group 1 and in adults, unimodal ISI effects were most prominent at fronto-polar recording sites in the first time window (90–150 ms (children) and 70–120 ms (adults), respectively). Beyond 260 ms post-stimulus, unimodal ERP refractory effects were obtained in all age groups (Figures 3, 5).

#### Crossmodal refractory period effects

In children aged 4–6 years, ERP amplitudes were significantly influenced by a modality switch, the preceding ISI and the recording site (see significant of marginally significant interaction of MT × ISI × topographical factor in the Five-Way- and Three-Way-ANOVA, Table 5). In the *post-hoc* analyses, crossmodal refractory effects were found over frontal, fronto-central, and central brain regions until 250 ms post stimulus (Figures 3, 5). In older children and in adults, the Five-Way- and the Three-Way-ANOVA revealed significant main effects of MT and/or ISI and interactions of MT × ISI × one or more topographical factors (see Tables 4, 6, and 7), which again indicated the influence of preceding modality, the preceding ISI and the recording site on ERP amplitudes. In contrast to the results of the *post-hoc* analyses in the youngest age group, crossmodal ISI effects were most pronounced over the posterior scalp after 150 ms post stimulus, and these effects were most reliable in adults (Figures 3, 5).

## Discussion

The present study tested whether uni- and crossmodal ERP refractory effects, indicating uni- and multisensory development, emerge sequentially (hierarchical developmental view) or parallel (differentiation developmental view). ERPs elicited in response to auditory and visual stimuli presented with two different ISIs (1000, 2000 ms) were analyzed as a function of the age of the participants, the modality of the preceding stimulus and the ISI.

ERP amplitudes to both visual and auditory stimuli were modulated by the modality of the preceding stimulus and the ISI. The degree of modulation depended on (i) the recording site and (ii) the age of the participant. The most pronounced crossmodal refractory effects both for visual and auditory ERPs were observed in the youngest age group (4–6 years). Crossmodal refractory period effects emerged earlier and with a different and broader topography in the youngest children compared to adults: They had a frontal and fronto-central topography irrespectively of stimulus modality in the youngest age group but were most pronounced over the posterior scalp in the oldest children and in adults. By contrast, unimodal refractory effects were observed for both modality conditions in all age groups.

We interpret our data in favor of the multisensory differentiation (Lickliter and Bahrick, 2004) and the multisensory perceptual narrowing account (Lewkowicz and Ghazanfar, 2009). By contrast, our results seem to be incompatible with the idea of a strict hierarchical development (Piaget, 1952), with unimodal processing maturing prior to the emergence of the first crossmodal interactions.

Firstly, we found the most prominent crossmodal interactions in the youngest age group (4–6 years). These effects emerged within the first analyzed time window (<150 ms). By contrast, crossmodal refractory effects in older children and adults were observed beyond the time epoch of 150 ms. These results suggest that at earlier processing stages, both in the visual and auditory processing pathway, crossmodal interactions occur in young children but not in older children and adults. Interestingly, these early crossmodal interactions had a fronto-central topography which was clearly distinct from the parietal topography of later crossmodal interactions in older children and adults. These developmental trends were observed irrespectively of the stimulus modality. It might be speculated that the early crossmodal interactions observed in children were due to still existing exuberant connections between sensory systems (Innocenti and Clarke, 1984). As development progresses, crossmodal interactions become more specific as a result of crossmodal experience (Lewkowicz and Ghazanfar, 2009). More specific, crossmodal interactions might emerge from the setting up of multisensory areas such as those in the parietal cortex (Bolognini et al., 2010; Kamke et al., 2012). It is important to note that these results are not incompatible with previous reports in adults demonstrating earlier (<100 ms) crossmodal ERP effects in adults (Giard and Peronnet, 1999; Molholm et al., 2002). These authors compared the processing of unimodal vs. crossmodal stimuli, while we presented unimodal stimuli only and assessed the refractoriness of different stages of the auditory and visual processing pathway due to a preceding stimulus.

Although there were some differences between age groups, mainly in topography, reliable unimodal refractory effects were observed in all groups of children, irrespectively of age and in adults. These results are in accord with Coch et al. (2005), who reported unimodal refractory effects both for auditory and visual ERPs in their youngest group as well (6–8 years). Thus, though the finding of an earlier maturation of uni- compared to crossmodal processing would be compatible with a hierarchical view of sensory development, the parallel finding of pronounced crossmodal interactions in the youngest but not older age groups is incompatible with this account. Earlier behavioral studies have reported that unisensory processing reaches adult level earlier than multisensory processing (Röder et al., 2013). Thus, uni- and multisensory processing seem to progress in parallel with a longer developmental time course of crossmodal (see Brett-Green et al., 2008; Brandwein et al., 2010) than unimodal development. Indeed, a number of recent studies has consistently shown a protracted developmental time course of multisensory processes into adolescents (Gori et al., 2008; Nardini et al., 2008; Barutchu et al., 2009).

Taken together, the present study provides evidence for the intersensory differentiation and multisensory perceptual narrowing approach to explain multisensory development. Some crossmodal interactions existing at earlier developmental ages seem to be eliminated and substituted by specific crossmodal interactions. Uni- and multisensory development runs in parallel with unimodal development leading.

### Conflict of interest statement

The authors declare that the research was conducted in the absence of any commercial or financial relationships that could be construed as a potential conflict of interest.
